# Genome-wide characterization of the GmLUX binding preferences and its epigenic features in the soybean genome

**DOI:** 10.3389/fpls.2025.1607224

**Published:** 2025-06-30

**Authors:** Lv Tianxiao, Hong Xiao, Jingxing Wang, Ling Zhang, Tian Fan, Mingkun Huang, Chang-En Tian, Hua Yang, Yufang Hu

**Affiliations:** ^1^ Guangdong Provincial Key Laboratory of Plant Adaptation and Molecular Design, Guangzhou Key Laboratory of Crop Gene Editing, Innovative Center of Molecular Genetics and Evolution, School of Life Sciences, Guangzhou University, Guangzhou Higher Education Mega Center, Guangzhou, China; ^2^ Suihua Branch of the Heilongjiang Academy of Agricultural Sciences, Suihua, China; ^3^ Jiangxi Provincial Key Laboratory of Ex Situ Plant Conservation and Utilization, Lushan Botanical Garden, Chinese Academy of Sciences, Jiujiang, China

**Keywords:** glycine max, GmLUX, ChIP-seq, multiple omics, epigenetics

## Abstract

Transcription factors function in complex regulatory networks to regulate various biological and physiological processes. In soybean (*Glycine max*), GmLUX, as an important component of the evening complex, plays a critical role in the regulation of soybean flowering regulation. In this study, the genome-wide characterization and epigenetic features of GmLUX binding sites have been analyzed using high-throughput sequencing methods, such as ChIP-seq, Hi-C, histone modification and ATAC-seq. In addition, combined with molecular experiments, GmLUX was found to be able to directly regulate the *CO-like* gene by facilitating chromatin interactions, suggesting a new regulatory pathway of GmLUX in controlling flowering, which provided the important genomic resources for a further understanding of its regulatory mechanism.

## 1 Introduction

Transcription factors (TFs) contribute significantly in regulating plant growth and development as well as the adaptation to the environment (e.g. stress responses, photosynthesis, specialized metabolite production) (Hrmova and Hussain, 2021; Strader et al., 2022; Dhatterwal et al., 2024; Gao and Dubos, 2024). Plant TFs carrying sequence-specific DNA-binding domains, particularly recognize and bind to specific DNA sequences (TF binding sites, usually with a typical short conserved consensus motif) to regulate the expression of associated genes as an activators or repressors (Liu et al., 1999; Blanc-Mathieu et al., 2024). The interactions between TFs and DNA typically disrupt nucleosome stability and elevate chromatin accessibility, which is partially responsible for actively engaged *cis*-regulatory elements (CREs) contained within accessible chromatin regions (ACRs) (Iwafuchi-Doi et al., 2016; Klemm et al., 2019; Ricci et al., 2019; Huang et al., 2022). ACRs with non-coding CREs are closely related to gene expression and TF-binding capacity (Klemm et al., 2019; Lu et al., 2019; Huang et al., 2022). Histone modifications are essential for regulating the chromatin accessibility, and flanking histone modifications indicate the transcriptional coregulators recruited to the ACRs (Klemm et al., 2019; Lu et al., 2019; Ricci et al., 2019; Zhou et al., 2021).

TFs play an important role in shaping plant development via regulating various downstream genes. As previously reported, soybean LUX transcription factor (GmLUX), a member of the evening complex, transcriptionally repressed the *E1* by binding to the LUX binding sites (LBS) of *E1s* promoters, which subsequently relieved the *E1s* suppression of two important *FT* genes (*FT2a* and *FT5a*) and promoted flowering (Lu et al., 2017; Bu et al., 2021). As a key TF, how GmLUX coordinated with other epigenetic signals to regulate downstream genes by interacting with genome-wide CREs remained elusive.

Here, in this study, we adopted the ChIP-seq method in combination with other high-throughput sequencing data, including Hi-C, histone modification and ATAC-seq, to genomically characterize the GmLUX (here referred to as Glyma.11G136600) binding sites and their epigenetic features in the soybean genome. Furthermore, by coupling these data and molecular experiments, we found that GmLUX could mediate the chromatin interaction to directly regulate a *CO-like* gene (Glyma.10G274300), which represents a new potential regulatory pathway of GmLUX. Together, these approaches provide important genomic resources for a comprehensive understanding of the regulatory mechanism of GmLUX.

## 2 Results

### 2.1 Genome-wide identification of GmLUX binding sites via ChIP-seq

**Figure 1:**
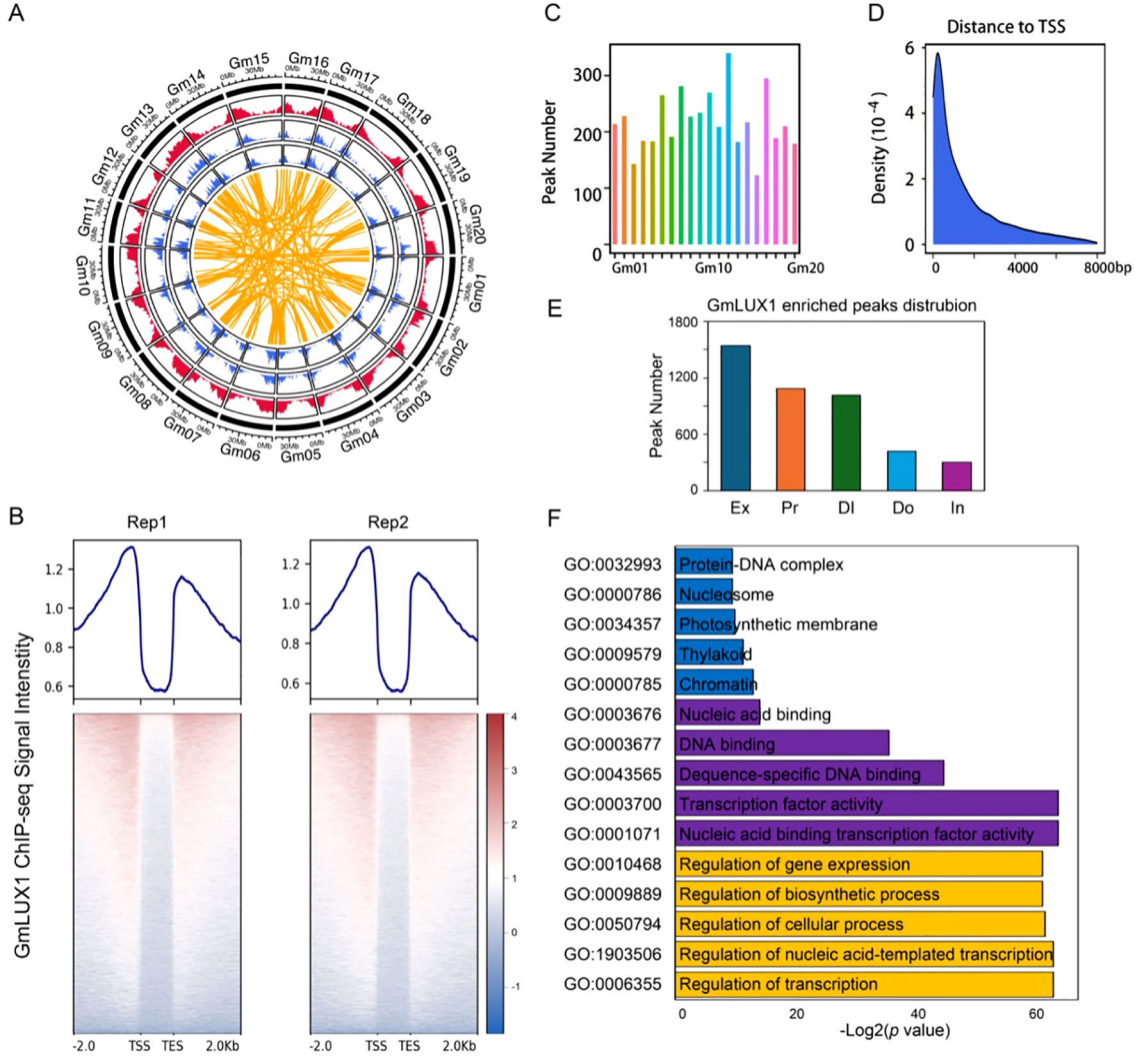
Data summary of GmLUX ChIP-seq data. **(A)** A circos digram showing the genomic distribution of GmLUX ChIPed peaks in the soybean genome. The red outer circle indicates the GmLUX associated genes, while the blue inner circles (two biological replicates) indicate the GmLUX enriched peaks. The yellow links indicate the homoeologous gene pairs targeted by GmLUX. **(B)** The GmLUX enriched profiles which have been normalized to input controls. Rep1 and Rep2 indicate two biological replicates. TSS, transcription start site; TES, transcription end site. **(C)** Number of GmLUX enriched peaks in each chromosome. **(D)** The distance from GmLUX binding sites to transcription start site (TSS) of their nearest associated gene. **(E)** Genomic distribution of GmLUX enriched peaks in soybean genome. Ex, exon; Pr, Promoter; DI, Distal intergenic region; Do, Downstream region; In, Intron. **(F)** Gene ontology (GO) enrichment analysis of the genes associated with GmLUX enriched peaks.

To gain deeper insight into the GmLUX (Glyma.11G136600) regulatory network in soybean, two replicates of GmLUX ChIP-seq were generated from leaves of *GmLUX-flag* transgenic plants to characterize the binding preference genome-wide (**Figure 1A**; **Supplementary Figure S1**). Up to 55 M reads were obtained for the ChIP-seq and input libraries (**Supplementary Table S1**). After alignment to the soybean reference genome (**Figure 1A**), a promoter and downstream enrichment pattern of GmLUX was observed (**Figure 1B**), and a total of 4,373 GmLUX binding peaks were identified by the MACS2 software (**Figure 1C**; **Supplementary Table S2**). Additionally, these peaks tended to be located within 2 kb from the transcription start site (TSS) of their corresponding nearest genes, such as the promoter and exonic regions (**Figures 1D, E**). Furthermore, the gene ontology (GO) enrichment analysis of the GmLUX peak-associated genes (3,312) (**Supplementary Table S2**) revealed several enriched GO terms, such as nucleic acid binding transcription factor activity (GO:0001071), transcription factor activity (GO:0003700) and sequence-specific DNA binding (GO:0043565), suggesting a robust transitional regulatory role of GmLUX (**Figure 1F**; **Supplementary Table S3**).

### 2.2 Construction of a putative GmLUX regulatory network

**Figure 2:**
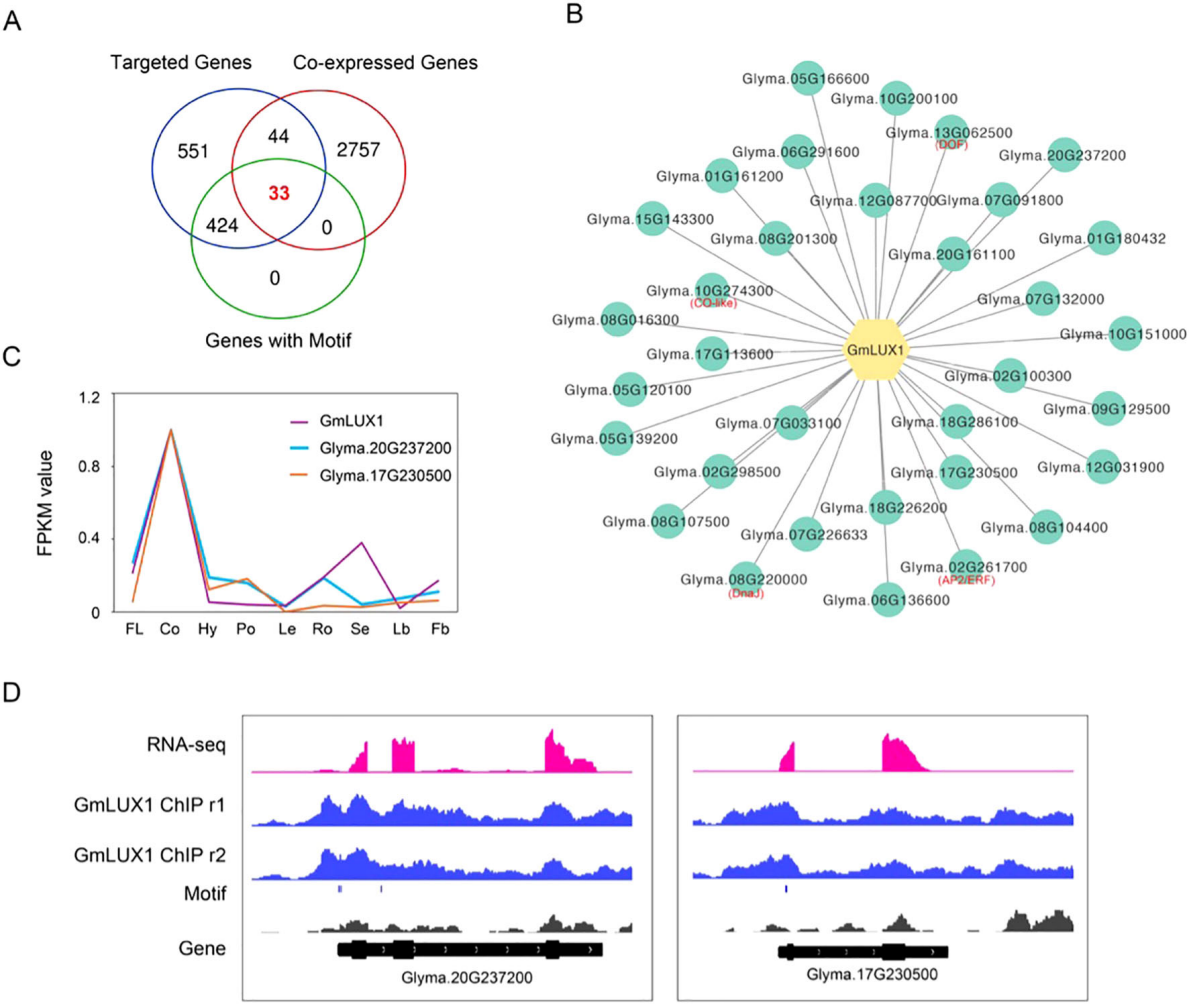
Putative network mediated by the GmLUX transcription factor. **(A)** Venn diagram showing the number of genes shared in the GmLUX targeted genes, GmLUX expression-correlated genes and genes harboring GmLUX binding motifs. **(B)** Construction of a proposed GmLUX regulatory network using the 33 shared genes in (2A). Four key TFs, including DOF, AP2/ERF, CO-like and DnaJ, are marked in red text. **(C)** Two examples of GmLUX co-expressed genes, Glyma.20G237200 and Glyma.17G230500. FPKM: fragments per kilobase of exon model per million mapped fragments. FL, flower; Co, cotyledon; Hy, hypocotyl; Po, pod; Le, leaf; Ro, root; Se, seed; Lb, leafbud; Fb, flower bud. **(D)** IGV screenshot showing two GmLUX targeted genes, Glyma.20G237200 and Glyma.17G230500. Glyma.20G237200 is a dormancy/auxin associated gene, and Glyma.17G230500 is a metallothionein like gene.

To further narrow down the potential genes directly targeted by GmLUX and constructed a GmLUX regulatory network, a comprehensive integration of GmLUX ChIP-seq, co-expression and motif scanning data was conducted, resulting in the identification of 33 key genes (**Figures 2A, B**; **Supplementary Table S4**). These genes, harboring a GmLUX binding motif in their promoters, were found to be co-expressed with GmLUX and associated with GmLUX enriched peaks, such as *Glyma.20G237200* and *Glyma.17G230500* (**Figures 2C, D**). Subsequently, these genes were then used to construct a putative GmLUX regulatory network with GmLUX as the central hub (**Figure 2B**). In addition, flower related genes (**Supplementary Table S2**) were identified in this network, such as *DOF* (Glyma.13G062500), *AP2/ERF* (Glyma.02G261700), *CO-like* (Glyma.10G274300) and *DnaJ* (Glyma.08G220000) (**Figure 2B**), which were reported to have potential roles in regulating flower and served as potential candidate genes for further investigation.

### 2.3 The epigenetic features of GmLUX binding peaks

**Figure 3:**
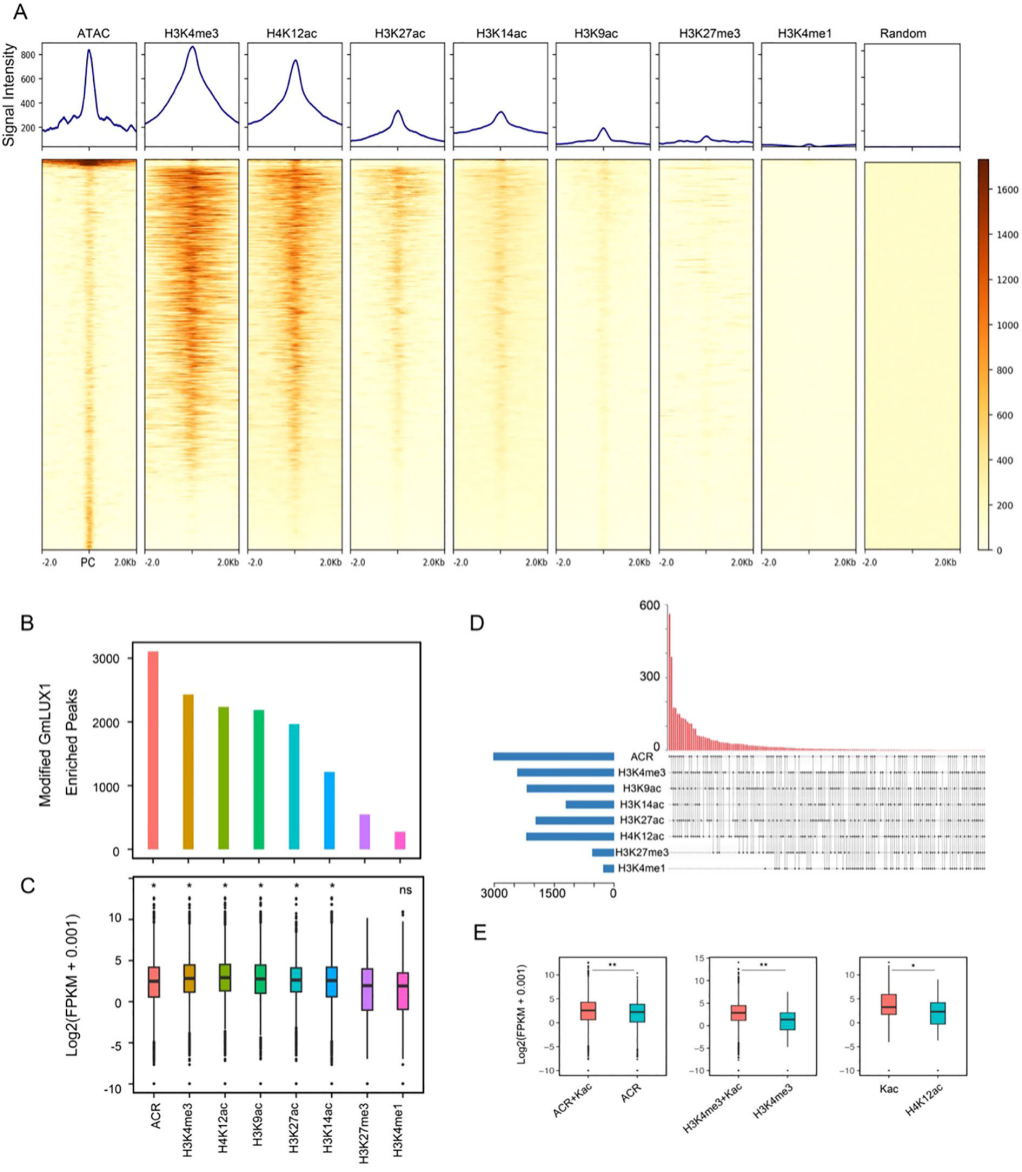
Epigenetic modifications at GmLUX binding peaks. **(A)** Enriched signals of ATAC-seq, H3K4me3, H4K12ac, H3K27ac, H3K14ac, H3K9ac, H3K27me3 and H3K4me1 ChIPed at the GmLUX peak center (PC), respectively. Random means the random genomic regions, which served as a control. **(B)** Number of GmLUX peaks associated with the distinct epigenetic modification signal. **(C)** Expression levels of genes associated with distinct modified GmLUX peaks. Asterisk (*) indicates the expression level is significantly higher than H3K27me3-modified GmLUX peaks associated genes (*p* < 0.05). Ns, not significant. *P* value was calculated by Wilcoxon test. ACR, accessible chromatin region. **(D)** Up-set Venn diagram showing the numbers of GmLUX enriched peak associated with multiple epigenetic modification signals. **(E)** The GmLUX targeted genes with multiple epigenetic modification signals are generally higher than those only with single modification signal. Two asterisks (**) indicate the *p* value less than 0.01, while one asterisk (*) means 0.01 < *p* < 0.05. Kac indicates four histone acetylation. *P* value was calculated by Wilcoxon test.

The epigenetic states of chromatin are usually closely associated with TF binding sites, serving as an additional layer of gene regulation (Cuellar-Partida et al., 2012). Hence, three histone modifications were generated by ChIP-seq, including H3K9ac, H3K27ac and H3K4me1; moreover, ChIP-seq data for four additional histone modifications (H3K27me3, H3K4me3, H4K12ac and H3K14ac) were downloaded, along with ATAC-seq data, to comprehensively understand the epigenetic states around GmLUX binding peaks (**Figure 3A**; **Supplementary Figures S2, S3**). As shown in **Figure 3A**, a strong enrichment of ATAC-seq, H3K4me3 and H4K12ac was observed at the center of GmLUX binding peaks, accompanied by a relatively weaker signal of H3K27ac, H3K14ac and H3K9ac, while little enrichment of H3K27me3 and H3K4me1 signals was observed at these regions. Consistent with these observations, we identified over 2,000 GmLUX binding peaks with ATAC-seq, H3K4me3 and H4K12ac modifications, and a few of peaks with H3K27me3 and H3K4me1 modifications (**Figure 3B**). Since H3K27me3 modification is a repressive mark, in contrast to the remaining active epigenetic marks, a significant difference was observed between the expression of H3K27me3-modified GmLUX binding peaks associated genes and those genes with other epigenetic mark modified peaks (**Figure 3C**). These observations indicated that GmLUX binding peaks associated with the active epigenetic marks (e.g. ATAC, H3K4me3 and H4K12ac) (**Supplementary Figure S5**) to regulate downstream gene expression.

Furthermore, many GmLUX binding peaks with multiple modifications were observed, which could have a significant impact on the downstream gene expression (**Figure 3D**; **Supplementary Table S5**). For example, gene expression with ATAC and acetylation (Kac) modified peaks was significantly higher than that with ATAC alone, and similar situations were also found in the H3K4me3&Kac versus H3K4me3 group, as well as Kac versus H3K12ac group (**Figure 3E**).

In addition, the epigenetic modification enrichment pattern of the distal GmLUX binding peaks was distinct from the proximal peaks, except for the ATAC-seq signal (**Supplementary Figure S4**). As the distal ATAC-seq signal was served as an indicator of potential distal enhancer-like elements, this observation suggested an enhancer role for distal GmLUX binding peaks, which required further investigation.

### 2.4 The impact of presence/absence of GmLUX binding peak on the expression of homologous genes

**Figure 4:**
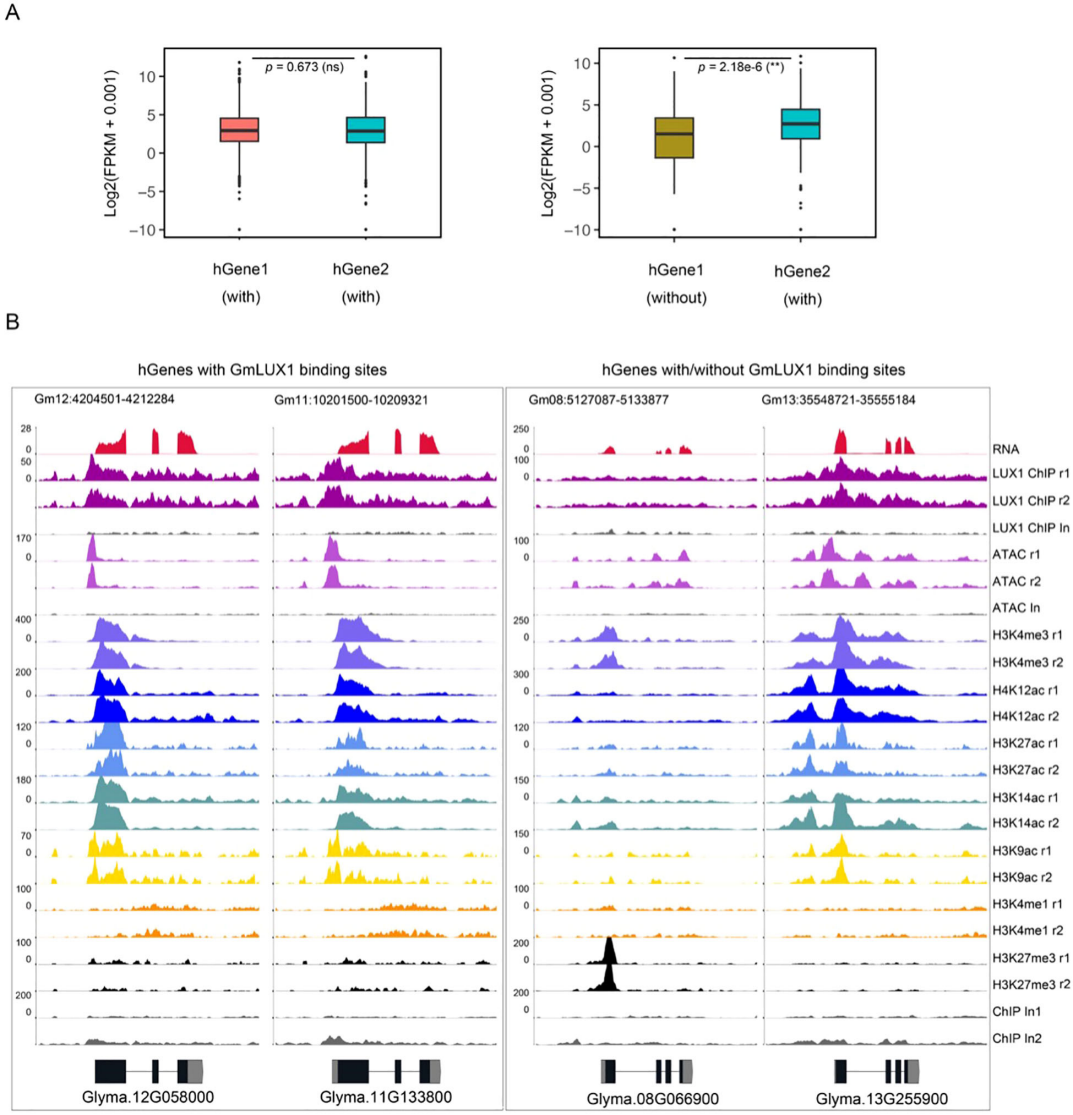
Potential effects of presence/absence of GmLUX binding region in homologous genes on gene expression. **(A)** Expression levels between homologous genes (hGenes) bound by GmLUX show no significant (ns), while the expression of genes associated with GmLUX is significantly higher than that of its homologous genes without GmLUX peaks. *P* value was calculated by the Wilcoxon test. **(B)** Two examples of hGenes with or without GmLUX binding peaks. Left panel, hGenes both with GmLUX have similar epigenetic modification signals; Right panel, distinct epigenetic modified signals are observed between hGenes with or without GmLUX binding peaks.

As a palaeopolyploid plant (Schmutz et al., 2010), soybean contains lots of duplicated genes in its genome. As previously reported, the gain and loss of *cis*-regulatory sequences (e.g. the ACR) could have a significant effect on the expression of homologous genes (hGenes) (Huang et al., 2021c; Fang et al., 2023). We focused on 1,011 genes with a promoter GmLUX binding site, identified their corresponding hGenes, and checked whether their hGenes shared the GmLUX binding peaks with them. Among these 1,011 paired hGenes, only 224 hGenes (22.2%) were bound by the GmLUX, while 787 hGenes (77.8%) exhibited an absence of GmLUX binding (**Supplementary Table S6**). Furthermore, it was observed that those hGenes with both GmLUX binding peaks had similar epigenetic modifications and showed no significant impact on gene expression (*p* = 0.673) (**Figures 4A, B**; **Supplementary Table S6**). However, the expression of those hGenes without GmLUX binding peaks was significantly lower than their hGenes (**Figures 4A, B**; **Supplementary Table S6**). In addition, the absence of GmLUX binding peaks also caused obvious changes in the epigenetic features of hGenes (**Figure 4B**; **Supplementary Table S6**). These data suggested that the presence or absence of *cis*-regulatory sequences during gene duplication were important for maintaining the expression of hGenes, which may have implications for the subsequent functionalization of these genes.

### 2.5 Molecular evidence for GmLUX-mediated promoter-enhancer interaction for gene expression

Enhancer elements played an important role in gene activation and could be located in the intergenic or intronic accessible regions (Zhu et al., 2015; Meng et al., 2021). In addition, as previously reported, TF could mediate the promoter-enhancer interaction to modulate gene expression in soybean (Huang et al., 2023). In this study, a total of 1,018 and 301 GmLUX binding peaks were observed located in the intergenic and intronic regions of the soybean genome, respectively (**Supplementary Table S2**). Of these, 837 intergenic and 160 intronic peaks overlapped with ATAC-seq peaks. Furthermore, a total of 114 genes were associated with multiple GmLUX binding peaks (41.8% associated with promoter and intronic GmLUX peaks and 58.2% associated with promoter and intergenic peaks) (**Supplementary Table S2**). Since ACRs identified by ATAC-seq served as an indicator of enhancer-like elements, this observation indicated that GmLUX was able to mediate enhancer-like elements to regulate gene expression.

**Figure 5:**
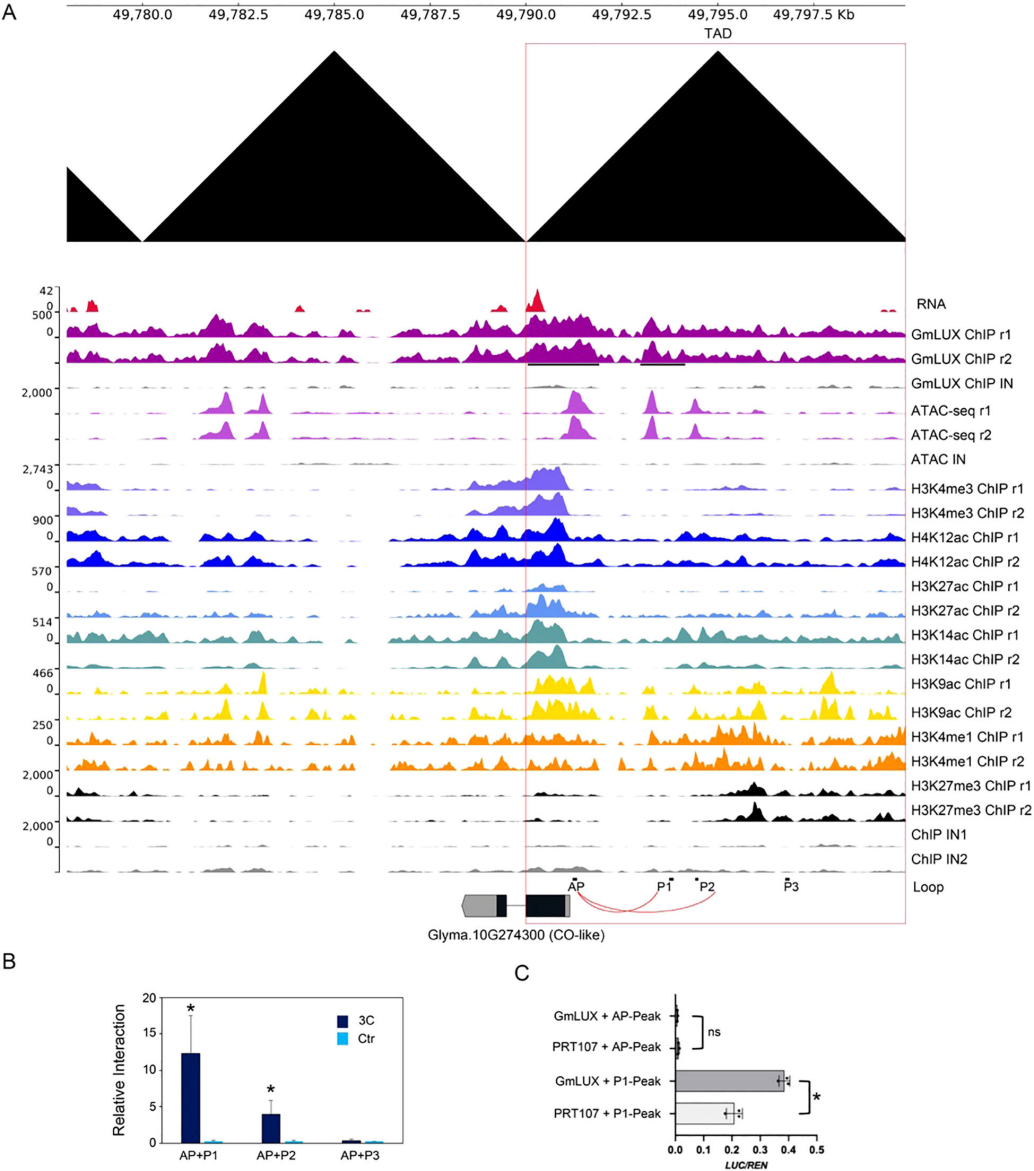
Potential promoter-enhancer loop mediated by GmLUX to regulate gene expression. **(A)** An example of promoter-enhancer interaction is validated by 3C-qPCR. The *CO-like* gene, Glyma.10G274300 is used as the example, bound by GmLUX at AP and P1 regions that shared overlapping with the ATAC-seq signal. The upstream of *CO-like* gene is located in a TAD domain (indicated in a black triangle). 3C-qPCR primers were design at P1, P2 and P3 region. Primers in AP was used as anchored primers. The DNA loop, AP-P1 and AP-P2 validated by the 3C-qPCR are indicated in red links. **(B)** Relative DNA amounts in 3C-qPCR. The relative 3C signal was normalized to an internal control, and the genomic DNA was used as the control template. Asterisks (*) indicates a significant change compared to the gDNA (*p* < 0.05, by Student’s *t-*test). **(C)** Transient experiments show that GmLUX can activate the P1 associated GmLUX enriched peaks (P1-peak) but not the AP associated peak (AP-peak). PRT107 is an empty vector used as a control. LUC, Firefly Luciferase. Ren, Renilla reporter for normalization. LUC/Ren indicates the relative activities. Asterisk (*) indicates the significant difference compared to the empty vector by Student’s *t*-test (*p* < 0.05). Ns, no significance.

To further test our hypothesis, a flowering-related gene, *CO-like* (Glyma.10G274300) was selected as a candidate for further validation. This *CO-like* gene harbored multiple *cis*-regulatory regions located in its upstream region (three peaks of ATAC-seq and two peaks of GmLUX) (**Figure 5A**). Interestingly, *CO-like* was associated with several active histone modifications (e.g. H3K4me3, H4K12ac, H3K14ac etc.) and had relatively high expression in soybean (**Figure 5**). Moreover, the upstream region of this *CO-like* gene located in a topologically associating domain (TAD, showed as a black triangle) region according to the Hi-C data, indicating a high frequency of chromatin interaction in these regions (**Figure 5A**). To confirm such an interaction, the 3C-PCR was performed using three primers (P1, P2 and P3) and an anchor primer (AP). It showed that the interaction signals of AP-P1 and AP-P2 were detected, except for that of AP-P3 (**Figure 5B**). Since AP and P1 were close to two GmLUX binding peaks, the data suggested that GmLUX mediated the AP-P1 interaction to regulate *CO-like* gene expression. Interestingly, in the transient experiments, only the P1 but not the AP *cis*-regulatory sequences had relatively high activities in protoplasts and could be activated by GmLUX overexpression (**Figure 5C**), indicating the distal binding region (e.g. P1) was necessary for GmLUX to regulate its target genes (e.g. the *CO-like* gene).

## 3 Discussion

Plant LUX is a SHAQYF-type GARP transcription factor containing a single MYB domain that can bind to the LBS motif (GATA/CCG) in target genes promoters (Hazen et al., 2005; Onai and Ishiura, 2005; Helfer et al., 2011; Zhang et al., 2019). In soybean, two LUX homologs were functionally redundant but together played critical roles in regulation of soybean flowering by directly binding to the LUX binding sites of *E1s* promoters (Lu et al., 2017; Bu et al., 2021; Lin et al., 2022). In this study, a multi-omics analysis has been adopted to identify a novel regulatory pathway of a GmLUX gene (Glyma.11G136600) that promotes soybean flowering.

In this study, the GmLUX ChIP-seq was performed using the robust, low-input requirement ChIPmentation method, which has been successfully validated in *Arabidopsis* (Lee and Bailey-Serres, 2019) and soybean (Huang et al., 2021b). Using this approach, the genome-wide distribution of GmLUX-specific binding sites has been determined, and a putative robust transitional regulatory network centered on GmLUX was also established. In this network, a *CO-like* gene (Glyma.10G274300) was identified, which has been reported to potentially regulate flowering (Wu et al., 2014; Cao et al., 2015), indicating the advantageous role of the multi-omics approach in mining the key regulatory factors involved in specific developmental stages.

Enhancer elements, essential cis-regulatory elements, can mediate the formation of chromatin loops to regulate gene expression (Li et al., 2019; Ricci et al., 2019; Huang et al., 2023). In this study, similar to the GmJAG1 (Huang et al., 2021b), we showed that GmLUX could mediate enhancer-like elements to regulate gene expression. This discovery is due to the relative ease of access to many high-throughput data (ChIP-seq, ATAC-seq and Hi-C etc.). For example, the high-throughput sequencing data, such as Hi-C, is necessary for the precise location of enhancer-like elements and the enhancer-mediated DNA loops on a genome-wide scale (Sanyal et al., 2012). Based on Hi-C analysis, a high frequency of sequence interactions has been observed between GmLUX and enhance-like elements of the CO-like gene which was associated with multiple active histone modifications and contained several cis-regulatory regions located in its upstream region (**Figure 5**). Also, to genome-wide map the enhancer-promoter interaction, new technologies such as the STARR-seq (Arnold et al., 2013; Zhang et al., 2022), HiChIP (Mumbach et al., 2016) etc., can be used for further investigation. Given the importance of non-coding regulatory sequences in gene regulation (Huang et al., 2021a) and the increasing amount of high-throughput sequencing data, a comprehensive database such as the ENCODE project focused on these data in soybean, is important for further investigation into the function of these non-coding elements.

Taken together, a flowering-related gene, *CO-like* can be directly regulated by GmLUX binding to its promoter, according to a comprehensive analysis of ChIP-seq, histone modification, Hi-C, ATAC-seq and molecular experiments, suggesting a new potential GmLUX regulatory pathway in modulating soybean flowering.

## 4 Method and material

### 4.1 Plant materials and growth conditions

*Glycine max* (L.) Merr. cultivar Williams 82 (W82) was used as the wild type in this study, and transgenic W82 with *35S:GmLUX-Flag* (Bu et al., 2021). Transgenic and wild-type seeds were sterilized with 75% ethanol and placed on sterilized vermiculite for germination, until their cotyledons were fully expanded. These seedlings were transferred to soil and grown in the greenhouse (16 h light/8 h dark, 25-28°C) to develop the healthy trifoliate leaves, which were utilized for subsequent experiments.

### 4.2 ChIP-seq library construction and data processing

The construction of GmLUX ChIP-seq library was followed the previous study (Huang et al., 2021b), with minor modifications. Briefly, about 0.5 g of leaves from *GmLUX-flag* transgenic plants were utilized for nuclear isolation. The chromatin fragments were pull-down by the anti-Flag antibody (Sigma). The ChIPed and input DNA used for library construction were processed by Tn5 transposase (Vazyme). Histone ChIP-seq library construction was followed the method (Huang et al., 2021b; Zhang et al., 2023) and the anti-H3K27ac, anti-H3K9ac and anti-H3K4me1 antibodies (Millipore) were utilized in this study. All the ChIP-seq libraries were sequenced on the PE150 mode of the Illumina platform.

The raw data of ChIP-seq were trimmed by TrimGalore (http://github.com/FelixKrueger/TrimGalore) and the filtered reads were mapped to the soybean reference genome (V4, https://phytozome.jgi.doe.gov) by the Bowtie2 (Langmead and Salzberg, 2012). The high-quality mapped reads (MAPQ > 30) were extracted by SAMtools and used for peak calling by MACS2 (Zhang et al., 2008; Li et al., 2009). For broad peak calling, the parameters were set as ‘-trackline -extsize 147 -broad -q 0.01 -nomodel -buffer-size 500000’, while the default parameters were used for narrow peak calling. The input library was used as a control. The profile of ChIP-seq is normalized to input controls by the BamCompare function in Deeptools (Ramírez et al., 2014). Enriched peaks annotation was performed via HOMER (http://homer.ucsd.edu/homer/). The visualization of ChIP-seq data was processed using the Deeptools (Ramírez et al., 2014), pyGenomtrack (Lopez-Delisle et al., 2021) or IGV (Thorvaldsdóttir et al., 2013) software.

### 4.3 RNA-seq data analysis

The RNA-seq data were obtained from a previous study (Huang et al., 2021b). Raw RNA-seq data were filtered by TrimGalore with the default parameters, and then mapped to the soybean reference genome using HISAT2 (http://daehwankimlab.github.io/hisat2). Gene expression levels were calculated by Cuffnorm (http://cole-trapnell-lab.github.io/cufflinks/cuffnorm) and represented by the fragments per kilobase per million mapped reads (FPKMs), which were utilized for subsequent analysis.

### 4.4 Hi-C data analysis

The leaf Hi-C data were downloaded from a previous report (Wang et al., 2021) and followed its analysis pipeline with minor modification. Briefly, the raw data was trimmed with the adaptors and were processed by the HiC-Pro (Servant et al., 2015) with default parameters. Seft-circle, dangling-end reads were removed, and the contacts were normalized by the iterative correction and eigenvector decomposition (ICE) method. Then the TAD domain was called by HiCexplore (Wolff et al., 2022) with default parameters and visualized by pyGenomtrack (Lopez-Delisle et al., 2021).

### 4.5 Motif analysis

The GmLUX motif (Motif ID: TFmatrixID_0354) position weight matrix (PWM) was downloaded from the PlantPAN database (Chow et al., 2019) and the FIMO from the MEME suite (Bailey et al., 2009) was used for motif scanning in the GmLUX peak region.

### 4.6 ATAC-seq data analysis

The soybean leaf ATAC-seq raw data were obtained from previous study (Huang et al., 2021b), and filtered by the TrimGalore and mapped to the reference genome via Bowtie2 (Langmead and Salzberg, 2012) with the parameters set as: –very-sensitive -X 1000. The mapped reads with a MAPQ value over 30 were used for peak calling via Genrich software on the ATAC mode (https://github.com/jsh58/Genrich). Visualization and annotation of ATAC-seq data was performed similarly to the ChIP-seq mentioned above.

### 4.7 Measurement of transcriptional activity

To confirm the transcriptional activation of GmLUX, the full-length CDS of GmLUX was cloned into the pRT107 vector which was derived by the 35S promoter, while the two GmLUX enriched peak regions associated with the CO-like gene were cloned into the pGreenII-0800-LUC vectors and confirmed by Sanger sequencing. Then the validated plasmids of pRT107-GmLUX and pGreenII-0800-GmCO-like-Luc were co-transformed into *Arabidopsis* protoplasts, isolated from the leaves of 3-week-old Col-0 by the PEG/CaCl2 method (Yoo et al., 2007). After 16 h of incubation at 22°C, the total proteins were extracted, and transcriptional activities were measured using the Dual-Luciferase Reporter Assay System (Promega, E1910) kit followed the manual instructions. The fluorescence value of firefly luciferase (LUC) and Renilla luciferase (Ren) was determined by Promega GloMax 20/20 system. The transcriptional activity of GmLUX against the CO-like associated peaks was evaluated by the ratio of LUC/Ren value. The experiments were performed in three biological replicates.

### 4.8 Chromosome conformation capture quantitative PCR

The 3C-qPCR was conducted according to the previous study (Hagege et al., 2007; Liu, 2017; Huang et al., 2023). Briefly, the isolated soybean leaf nuclei were digested with DnpII (NEB), and the sticky ends were filled with dWTP (W: A, T, G), biotin-14-dCTP (Sigma) and 40 U Klenow fragment (NEB). The blunt ends were then ligated with the T4 DNA ligase (NEB) following the manufacturer’s protocol. The biotin-labeled 3C-DNA was extracted by MyOne™ Streptavidin C1 Dynabeads (Invitrogen) according to the manufacturer’s protocol and used for qPCR using the TB Green Premix Ex II (Takara). The primers involved in 3C-qPCR analysis were listed in **Supplementary Table S1**.

## 5 Conclusions

In this study, the genomic characteristics of GmLUX (Glyma.11G136600) binding sites and their epigenetic features were revealed, using a modified ChIPmentation method, along with other high-throughput sequencing data, including Hi-C, histone modification ChIP-seq and ATAC-seq. Moreover, a new regulatory pathway of GmLUX has been identified, in which GmLUX facilitates the chromatin interaction to directly regulate a *CO-like* gene (Glyma.10G274300). This will provide important genomic resources for a comprehensive understanding of the regulatory mechanism of GmLUX.

## Data Availability

The data presented in the study are deposited in the NGDC (https://ngdc.cncb.ac.cn/) repository, accession number PRJCA034927.

## Supplementary material

Supplementary Figure 1 Pearson correlations between two replicates of GmLUX ChIP-seq. R1 and r2 indicate two biological replicates.

Supplementary Figure 2 Pearson correlations between two replicates of histone ChIP-seq data. R1 and r2 indicate two biological replicates. The H3K27me3 ChIP-seq data were downloaded from the previous study.

Supplementary Figure 3 Histone ChIP-seq enrichment analysis. H3K27ac, H3K4me1 and H3K9ac ChIP-seq signals are positively correlated with gene expression levels. TSS, transcription start site; TES, transcription end site; H, highly expressed genes (FPKM > 10); M, middle expressed genes (1 < FPKM ≤ 10); L, low expressed genes (FPKM ≤ 1); r1 and r2 indicate the two ChIP-seq replicates.

Supplementary Figure 4 Distinct enrichment pattern epigenetic modification between proximal and distal GmLUX enriched peaks. Proximal GmLUX peaks indicate those located near the gene region including promoter, exon, intron and downstream, while the distal peaks indicate the distance from peaks to nearest genes over 2 kb.

Supplementary Figure 5 Venn diagram showing the overlap of GmLUX bound peaks associated with ACR or H3K4me3/H3K12ac. P value was calculated by the hypergeometric test.
