# Supplementary figures and images for: Genome-wide characterization of the GmLUX binding preferences and its epigenic features in the soybean genome
Source: Front Plant Sci. 2025 Jun 30;16:1607224. doi: 10.3389/fpls.2025.1607224 (PMC12256510; doi:10.3389/fpls.2025.1607224)

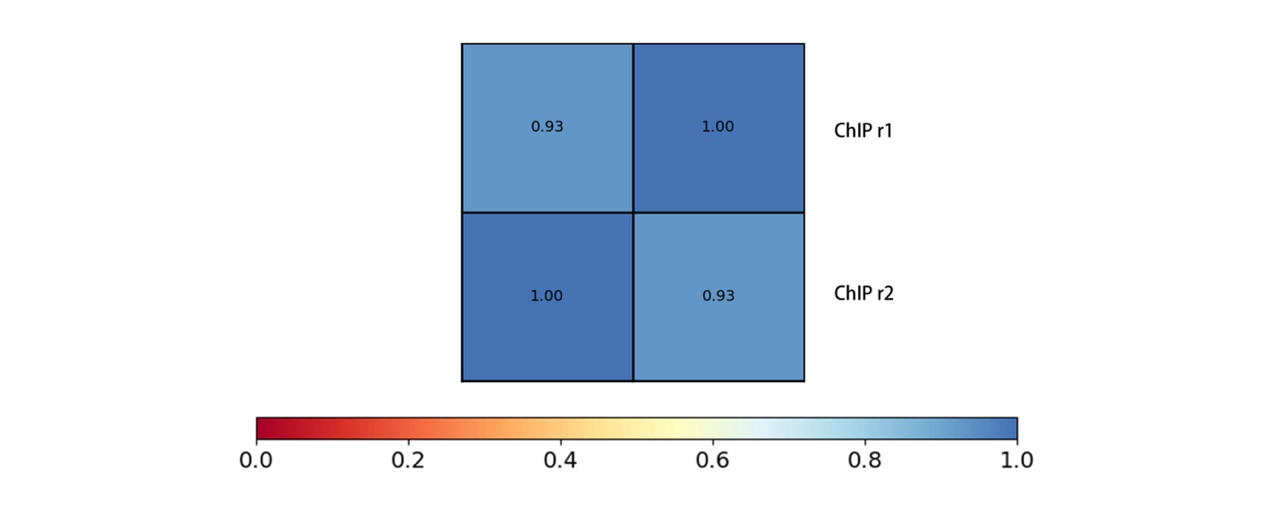

Supplement: Supplementary Figure 1 — Pearson correlations between two replicates of GmLUX ChIP-seq. R1 and r2 indicate two biological replicates. [file Image1.jpeg]

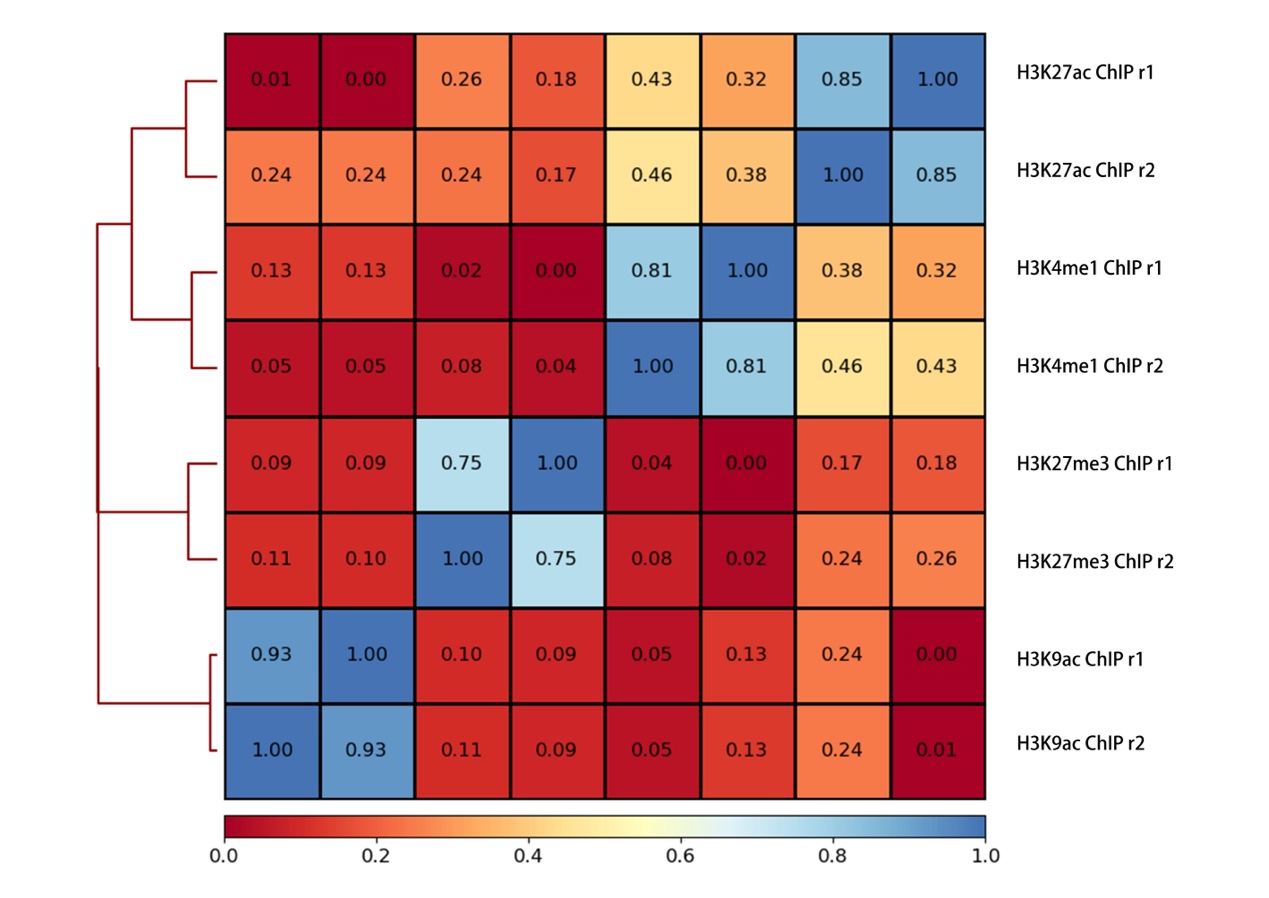

Supplement: Supplementary Figure 2 — Pearson correlations between two replicates of histone ChIP-seq data. R1 and r2 indicate two biological replicates. The H3K27me3 ChIP-seq data were downloaded from the previous study. [file Image2.jpeg]

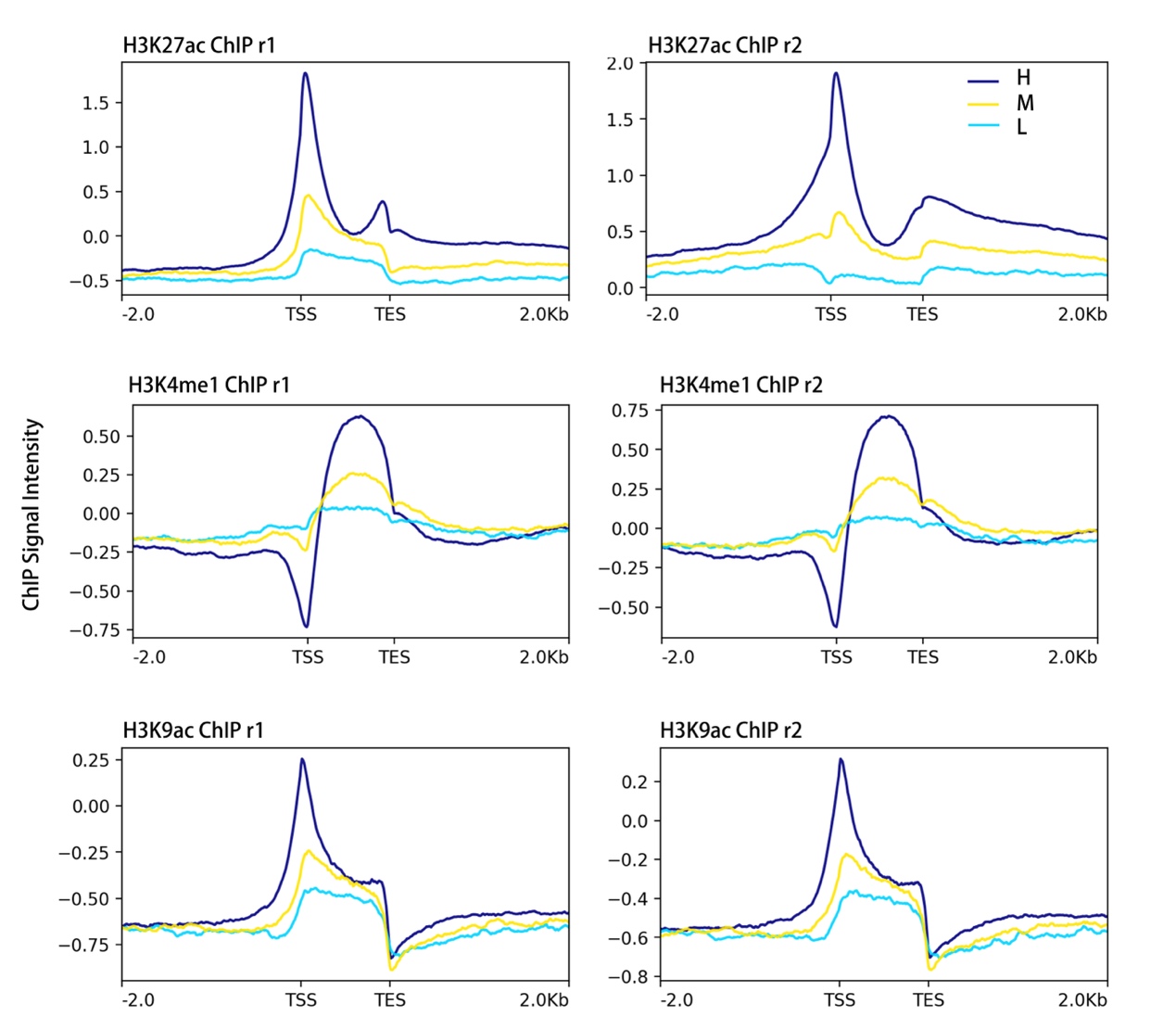

Supplement: Supplementary Figure 3 — Histone ChIP-seq enrichment analysis. H3K27ac, H3K4me1 and H3K9ac ChIP-seq signals are positively correlated with gene expression levels. TSS, transcription start site; TES, transcription end site; H, highly expressed genes (FPKM > 10); M, middle expressed genes (1 < FPKM ≤ 10); L, low expressed genes (FPKM ≤ 1); r1 and r2 indicate the two ChIP-seq replicates. [file Image3.jpeg]

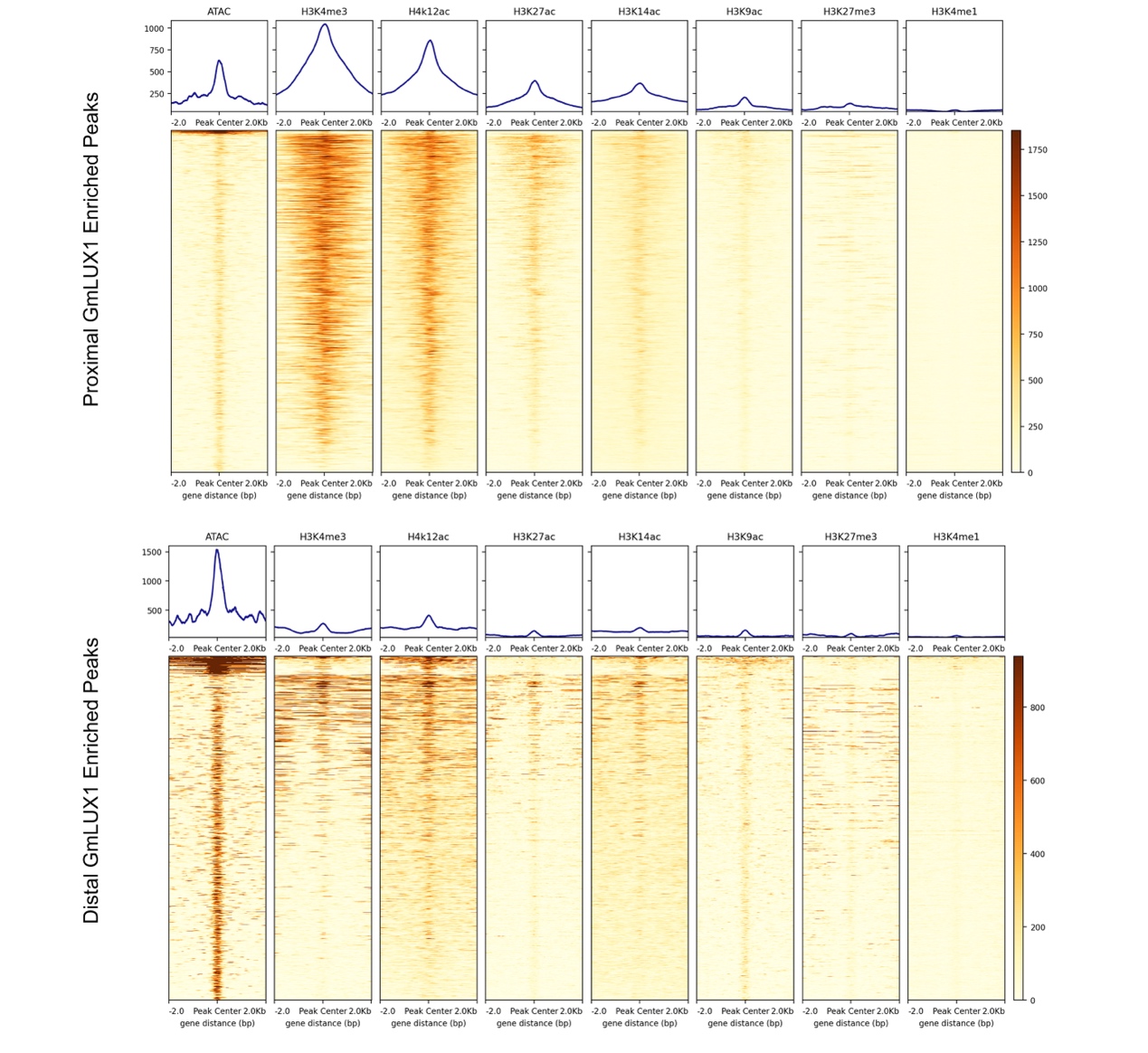

Supplement: Supplementary Figure 4 — Distinct enrichment pattern epigenetic modification between proximal and distal GmLUX enriched peaks. Proximal GmLUX peaks indicate those located near the gene region including promoter, exon, intron and downstream, while the distal peaks indicate the distance from peaks to nearest genes over 2 kb. [file Image4.jpeg]

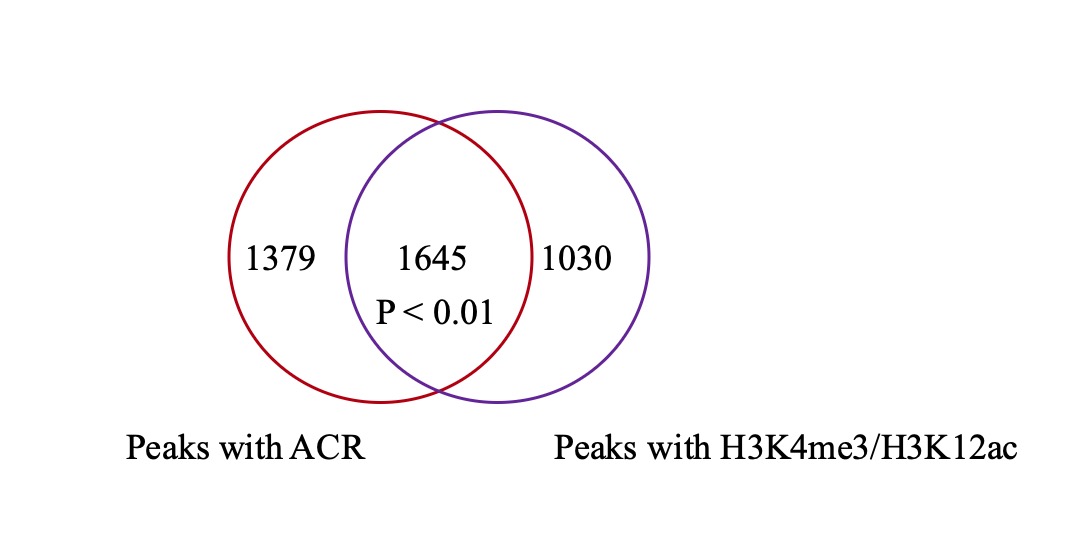

Supplement: Supplementary Figure 5 — Venn diagram showing the overlap of GmLUX bound peaks associated with ACR or H3K4me3/H3K12ac. P value was calculated by the hypergeometric test. [file Image5.jpeg]
